# Supplementary material for: Protein undernutrition reduces the efficacy of praziquantel in a murine model of Schistosoma mansoni infection
Source: PLoS Negl Trop Dis. 2022 Jul 15;16(7):e0010249. doi: 10.1371/journal.pntd.0010249 (PMC9328564; doi:10.1371/journal.pntd.0010249)
Supplement: Attachment — Submitted filename: Response to reviewers.docx [file pntd.0010249.s001.docx]

**RESPONSE TO REVIEWERS**

**Manuscript PNTD-D-22-00195**

**Manuscript title: Protein undernutrition reduces the efficacy of praziquantel in a murine model of *Schistosoma mansoni* infection**

**REVIEWER 1**

Fassi et al have studied the effect of protein undernutrition on the efficacy of praziquantel treatment on *S. mansoni* infection. The authors find that PZQ efficacy is reduced as consequence of protein undernutrition in experimental model of murine schistosomaisis. This is a highly relevant research question populations in which *S. mansoni* infection is endemic, often also suffer from malnutrition, including insufficient protein intake. Knowing how PZQ efficacy is affected by host nutritional status is therefore highly relevant. Although the experimental approach is generally sound, the way the manuscript is written and data are presented make it hard to read. This should be improved. Specifically, I have the following comments:

**Answer**

Thank you for your review. We are honoured to receive your appreciation and your valuable comments.

**Major comments**

1) The results section lists the main differences observed between the different experimental groups. However, it is now very difficult to read because it contains no rationale why certain parameters are analyzed. In addition, no summarizing sentences present at the end of each section, to bring across the main points of each of the findings. Also the headings of each section should reflect the findings of that section rather than what is measured. So, for instance, not ‘worm burden and egg load….in mice with a protein deficient diet and treated with PZQ’ but ‘PZQ treatment is less efficacious in reducing worm burden etc in Sm infected mice on a protein-poor diet’. These things should be changed, to improved readability.

**Answer**

Thank you for your remarks.

Many parameters have been measured in this study because the association between undernutrition and *S. mansoni* infection interferes with the parasitological outcome of the host (worm and egg burdens, enlargement of organs), its growth (body weight, Lee index), its haematology (red and white blood cells count), its general and lipid metabolism (glucose, protein, albumin, iron, calcium, total cholesterol, HDL, LDL….), its liver function (biomarkers of the liver function as transaminases, GGT…., oxidative stress markers as MDA and antioxidants, and histomorphometry) and its immune response (cytokines, chemokines and their gene expression).

Sections headings and summarizing sentences have been added in the results section. The headings of each section have been corrected to reflect the main findings (lines 233, 287, 312, 343, 376, 401, 439, 474, 523, 558 and 601). In addition, at the end of each result section, summarizing sentences have been added (lines 247, 271, 295, 298, 326, 358, 385, 412, 457, 505, 540, 573, 581 and 614)

2) The results section starts off with 10 tables, which are much harder to read then bar graphs. So please change them into bar graphs and display individual mice as dots, to be able to gauge inter experimental variation.

**Answer**

The results section begins with Table 4. Then Tables 4 to 9 have been changed to bar graphs with individual mice as dots. To harmonize all the manuscript figures, individual mice have also been plotted as dots in figures 1 to 4. The results section finally comprises 12 figures and 1 table.

3) What was the rationale to treat the mice with PZQ at day 36 post-infection, which is the prepatent phase, before egg deposition? In clinical settings, treatment will most likely take place in infections where egg-laying has already started.

**Answer**

According to Lenzi, 1998, couples of *S. mansoni* are observed in the mesenteric veins of infected mice from the 27^th^ day post-infection, oviposition started between 30^th^ and 34^th^ day post-infection and mature eggs are present in the liver and the intestine between 34^th^ and 36^th^ day post-infection (Lenzi JA**.** *Estudo do modelo Calomys callosus Renger, 1830 (Rodentia: Cricetidae) na infeção por Schistosoma mansoni Sambon, 1907. Parâmetros parasitológicos e histopatológicos*. Tese de Doutorado. Universidade Federal da Bahia, Salvador, Brasil, 1998).

Based on Lenzi’s findings, we began to treat mice in this study on day 36^th^ post-infection when egg-laying had already started, and the treatment lasted 28 days.

4) Where the mice randomized based on weight before infection? It doesn’t seem so, as there were some weight difference between the groups before Sm infection (table 4). This may have skewed some of the results.

**Answer**

Before *S. mansoni* infection, the mice in all groups considerably gained weight, and the body weight variation was not statistically different between all the groups of mice before the infection and even before the treatment, except for the group of infected and untreated mice receiving the standard diet (IN group) (see Fig. 1).

5) The authors attribute difference in PZQ treatment efficacy to differences in protein intake. However, before Sm infection, there seems to be a weight difference between the two groups with different diets. Hence differences in efficacy may in fact be attributed to initial weight differences, rather than differences in protein intake specifically. This could be have solved by performing paired feeding experiments, where caloric intake of the mice on a protein poor diet was matched by those on a standard diet. This should at least be discussed and mentioned as limitation of the study.

**Answer**

The body weight variation between the groups of mice with different diets, the standard diet (HN, IN and IN-PZQ groups) and the low-protein diet (HLP, ILP and ILP-PZQ groups) was not statistically significant (see Fig. 1). Thus, the difference in PZQ treatment efficacy could not be linked to initial weight differences between some groups of mice.

6) Please also measure type 2 cytokines in figure 3.

**Answer**

The opportunity offered to us for cytokine assay allowed us to explore only the mRNA expression of IL-13 as a Th2 cytokine. Therefore, the primer has been added in Table 3 (line 209), and the results are included in the text (lines 531 - 533) and Fig. 9.

7) What about hydroxyproline levels in the livers of these mice as a measure of fibrosis?

**Answer**

Effectively, hydroxyproline is an undeniable biomarker of fibrosis. We would have liked to measure it, but unfortunately, we could not get liquid nitrogen for the assay. However, we indirectly assess the liver fibrosis via collagen deposition on the liver parenchyma during histology study using the picrosirius staining (Fig. 10 and lines 566 to 571).

**Minor comments**

1)    The authors use the term ‘protein-deficient diet’. However, the type of diet used in this study is reduced in protein content, not deficient. This should be corrected throughout the manuscript

**Answer**

Thank you for your suggestion. The term “protein-deficient diet” has been changed to “low-protein diet” throughout the document.

2)    Please explain in the results section what the Lee index entails. This is explained later on, but should be explained at time of introduction

**Answer**

Lee index has been introduced and defined in the result section (Fig. 1 and line 244)

3)    Abbreviations of the experimental groups don’t have to be introduced in every single results section. Once at the beginning of the results section is enough

**Answer**

These remarks have been taken into consideration. We have reduced the use of the abbreviations of the experimental groups in the results section.

**REVIEWER 2**

Inadequate feeding is common in areas of poor sanitation where diseases such as schistosomiasis prevail. Kwashiorkor, a severe form of malnutrition common in some of these countries argue for the clear lack of protein-sufficient diet in these countries.

In this report, Jatsa and collaborators assessed the impact of protein-deficient diet on the efficacy of anti-schistosomiasis treatment with Praziquantel.

Using a mouse model of infection, the authors fed young mice (<5 weeks old) with protein-sufficient or protein-deficient diets for 5-6 weeks and infected them individually with 50 S mansoni cercariae. Animals were treated for 5 consecutive days with 100 mg/kg of PZQ after 6 weeks of infection and then culled after 9 weeks of infection to retrieve parasitological, pathological, biochemical and immunological markers of infection.

The authors report on a reduced ability of PZQ to clear parasites, restore ion levels, improve pathology and ameliorate stress in S mansoni-infected and PZQ treated protein-deficient diet fed mice whereas PZQ treatment in S mansoni infected mice and initially fed with protein-sufficient diet displayed reduced parasitological burden, ameliorated liver pathology and reverse immunomodulatory markers that are pathognomonic of s mansoni infection.

The authors went on to conclude on the possible negative influence of a protein-deficient diet on the schistosomicidal, antioxidant, anti-inflammatory and immunomodulatory activities of PZQ.

**General Comments**

The report is conceptually novel in its approach and does attempt to tackle a clearly relevant facet of schistosomiasis control in poor areas where undernutrition is common. As such the potential in providing preclinical insights on the current impact of undernutrition on PZQ treatment efficiency during schistosomiasis is clear and a novel addition to the current body of literature. The massive amount of data and the comprehensive appraisal of the differences in the experimental set-ups do considerably strengthen the robustness of the present report.

However, some points of concern are to be raised here to provide more clarity to the conclusions made by the authors and maximize the impact of the present report.

**Answer**

Thank you for your review. We are honoured to receive your appreciation and your valuable suggestions and comments.

1) The present report of body weight change in table 4 is quite cryptic and should be rather displayed in a graph (XY, linear regression option on graph pad). Having performed the same with the data provided by the authors in table 4, I did fail to clearly see the claimed difference. In fact, weight loss was not to be found for all infected animals as claimed by the authors, especially for PZQ treated groups at day 106 i.e. prior to treatment per se. The data do not support a reversal of weight loss but perhaps a prevention of weight loss, as presented. Perhaps a body weight variation curve (i.e. following up the changes of an animal body weight and plotting that over time) would be more informative. As it now stands, the clear baseline differences in body weight do preclude a conclusive analysis of the extent of body weight change per group

**Answer**

Thank you for your suggestions that have been considered. We calculated the body weight variation in each group of mice and displayed it in more informative curves (see Fig. 1). It is now clear that there is no change in the body weight variation between groups of mice until the beginning of PZQ treatment.

2) Lines 109-112:’ All procedures in this study followed the principles of laboratory animal use and care of the “European Community” guidelines (EEC Directive 2010/63/EEC) and were approved by the “Animal Ethical Committee” of the Laboratory of Animal Physiology of the Faculty of Sciences, University of Yaoundé I – Cameroon.’ The authors should provide an Ethics approval number for the study as is commonly assigned by established animal ethics committee.

**Answer**

The “Animal Ethical Committee” of the Laboratory of Animal Physiology of the Faculty of Sciences, University of Yaoundé I validate the research protocol of this study. The application for the deliverance of an ethical clearance has been deposited to the “Joint Institutional Review Board for Animal & Human Bioethics” (JIRB) of the “Centre for Research and Graduate Studies in Life Health & Environment Sciences”, University of Yaoundé I. We are still waiting for the Ethical clearance number.

3) Similarly, to the body weight in table 4, the organ weight indexes in table 5 would benefit from a bar graph representation of the different indexes coupled with a conclusive curve on the percentage change from naïve to infected and from infected to treated. It is paramount here to consider the reference to the naïve setting towards which the PZQ treatment should move the system rather than simply claiming differences induced on the infected by the PZQ treatment (IN-PZQ vs IN compared to ILP-PZQ vs ILP). The latter approach, taken by the authors, is misleading as it is clear that infection does not similarly affect the IN and the ILP mice when compared to their naïve controls HN and HLP respectively.

**Answer**

We also changed Table 5 into a bar graph and added the percentage change of organ indexes on top of each bar (see Fig. 2).

We compared PZQ-treated infected mice (IN-PZQ) to the naïve mice (HN) in the results section (lines 268 - 270).

Although the IN mice were more affected by the infection than the ILP mice, results from this study demonstrated that the liver and intestine weights of *S mansoni*-infected and PZQ treated low-protein diet-fed mice (ILP-PZQ) were not normalized as they were closed to those of their infected controls (ILP) (line 270 to 272)

4) Table 6 should also be displayed as bar graph for clarity. It is also necessary here to plot a variation curve to show how different the efficacy of PZQ is in one setting over the other i.e. a bar graph of worm burden changes after PZQ treatment in each group. This is the most critical result here, and should be more clearly represented to convey the key message of the paper.

**Answer**

Thank you for your suggestion that was taken into consideration. We displayed Table 6 as a bar graph and plotted the percentage change of worm burden and egg load on top of each bar (see Fig. 3).

5) Table 7-10 should be modified into graphs also i.e. presenting the data as bar graph and then performing statistical comparisons between HN vs HLP (what the diet does); HN vs IN & HLP vs ILP (what the infections does in the groups and plotting the changes here from infection in N vs infection in LP); IN-PZQ vs IN and vs HN compared to ILP-PZQ vs ILP and vs HLP (what treatment does and how close back to naïve does it bring the system) etc. PS: Note that table 10 is too large and truncated as it was inserted in the wrong orientation.

**Answer**

Tables 7 - 9 have been changed to bar graphs (see Figs. 4 – 6), while table 10 has been maintained (Table 4 in the new manuscript) and inserted in the good orientation (landscape mode).

The comparisons pointed out in this comment have already been performed using the multiple comparison Tukey post-test. We used different acronyms to express significant differences between groups (see the text of each section and figures captions) and where differences were not significant, we did not mention them on figures to avoid overloading. For example, a comparison between HN and HLP revealed a reduced AST activity in HLP mice (lines 315 – 317).

6) Figure 6: How do the authors explain such an observation; secondly, hepatic granuloma volumes are apparently reduced by PZQ treatment in IN-PZQ but not in ILP-PZQ whereas both do present a drastic abrogation of IL-13 production (figure 2), a key cytokine, along with IL-4, in granuloma formation. How could that be reconciled ?

**Answer**

We have made the same observation, and we correlated it to the elevated IL-5 serum level in ILP-PZQ mice compared to IN-PZQ mice. In fact, IL-5 promote fibrosis in the lung, liver and skin through the activation, differentiation and recruitment of eosinophils which activate myofibroblasts, macrophages and neutrophils that participate in fibrosis (Gharaee-Kermani *et al.,* 1998; Reiman *et al.,* 2006). Therefore, it can be a possible justification for the non-reduction of hepatic granuloma volume in ILP-PZQ mice.

7) Lines 736-737: The authors refer to an eventual decrease of fibrosis in their assays following PZQ treatment. Was this decline quantitatively reported in the figures (area coverage in liver sections or hydroxyproline per trapped eggs in tissue etc…) ? Also, was this fibrosis development affected in ILP when compared to IN mice ?

**Answer**

We would have liked to measure the liver hydroxyproline, but unfortunately, we could not get liquid nitrogen for the assay. However, we indirectly assess the liver fibrosis via collagen deposition on the liver parenchyma during histology study using the picrosirius staining (Fig. 10 and lines 566 to 571).

As we did not quantify hydroxyproline, we cannot objectively compare the fibrosis development in IN and ILP mice.

8) Does protein undernutrition affect the course of the disease + host responsiveness to the disease and/or does it affect the efficacy of PZQ? The protein undernutrition could very well impair the host immune system, a key actor in the killing process of worms by PZQ. In fact, after worm paralysis induced by the drug, the host immune system might play a critical role in finalizing the schistosomicidal activity of the treatment (Modha J, Lambertucci JR, Doenhoff MJ, McLaren DJ: Immune dependence of schistosomicidal chemotherapy: an ultrastructural study of Schistosoma mansoni adult worms exposed to praziquantel and immune serum in vivo. Parasite Immunol. 1990, 12 (3): 321-334. 10.1111/j.1365-3024.1990.tb00958.x.). This should be better discussed and the a more inclusive view incorporating this aspect should be reflected in the entire discussion.

**Answer**

This study did not reveal that the 14.60% low-protein diet interferes with the host immune response (lines 477 – 480). However, the low protein diet impairs calcium homeostasis by reducing its concentration in HLP mice (lines 346 - 347). Given the fact that rapid calcium influx in schistosomes leads to paralysis and death, a low calcium concentration in the worm microenvironment may contribute to its low death rate. Therefore, we can argue that the 14.60% low-protein diet interferes with PZQ efficacy. We discuss this issue in the document (lines 683 – 693).
